# Supplementary material for: Content-rich biological network constructed by mining PubMed abstracts
Source: BMC Bioinformatics. 2004 Oct 8;5:147. doi: 10.1186/1471-2105-5-147 (PMC528731; doi:10.1186/1471-2105-5-147)
Supplement: Additional File 2 — The original results of the above study (non-essential files are deleted to keep the file size under the limit set by BMC bioinformatics). [file 1471-2105-5-147-S2.bz2 › chilibotAdditionalFile2/dip10/768ID10633096E18159/html/FECA_FECR.html]

 


 **FECA** and **FECR** 
  
Found 16 abstracts in PubMed, retrieved 10.  
 

 What does Google say? 
 PDF only 
| .edu only 

---

**Interactive relationship** (e.g. stimulation, inhibition, etc)

**Neutral relationship**- Sites of Interaction between the  **FecA**  and  **FecR**  Signal Transduction Proteins of Ferric Citrate Transport in Escherichia coli K 12.  Ref: 12813067 J Bacteriol, 2003
- These data clearly support a model that proposes interaction of the periplasmic N terminus of  **FecA**  with the periplasmic C terminal portion of  **FecR**  and interaction of the cytoplasmic N terminus of  **FecR**  with FecI, which results in FecI activation.  Ref: 10633096 J Bacteriol, 2000
- Surface signaling in ferric citrate transport gene induction interaction of the  **FecA** ,  **FecR** , and FecI regulatory proteins.  Ref: 10633096 J Bacteriol, 2000
- In the same C terminal region, amino acid residues important for the interaction of  **FecR**  with  **FecA**  were identified by random and site directed mutagenesis.  Ref: 12813067 J Bacteriol, 2003
- **FecA**  1 79 interacts with  **FecR**  101 317 and  **FecR**  1 85 interacts with FecI 1 173.  Ref: 10633096 J Bacteriol, 2000
- The amino proximal end of  **FecA**  interacts with the carboxy proximal end of  **FecR**  in the periplasm.  Ref: 12732308 Curr Opin Microbiol, 2003
- The N terminal region of  **FecA**  was localized in the periplasm, which indicates that  **FecA**  probably interacts with  **FecR** , which is involved in signal transduction across the cytoplasmic membrane.  Ref: 9044267 Mol Microbiol, 1997
- Binding of ferric citrate to the outer membrane protein  **FecA**  initiates a signal that is transmitted by  **FecR**  across the cytoplasmic membrane into the cytoplasm where FecI, the sigma factor, is activated.  Ref: 10633096 J Bacteriol, 2000
- In in vitro binding assays,  **FecR**  that was His tagged at the N terminus His 10  **FecR**  and bound to a Ni nitrilotriacetic acid agarose column was able to retain  **FecA** , and  **FecR**  that was His tagged at the C terminus  **FecR**  His 6 retained FecI on the column.  Ref: 10633096 J Bacteriol, 2000
- **FecR**  receives the signal in the periplasm from the outer membrane protein  **FecA**  loaded with ferric citrate, transmits the signal across the cytoplasmic membrane, and converts FecI in the cytoplasm to an active sigma factor.  Ref: 12813067 J Bacteriol, 2003
- The data showed that  **FecR**  interacts in the periplasm with  **FecA**  to confer ferric citrate induced transcription of the fec transport genes and identified sites in  **FecR**  and  **FecA**  that are important for signal transduction.  Ref: 12813067 J Bacteriol, 2003
- We propose that  **FecR**  spans the cytoplasmic membrane once, interacts in the periplasm with its C terminal region with  **FecA**  occupied by ferric citrate, and transmits the information through the cytoplasmic membrane into the cytoplasm, where it converts FecI into an active sigma factor.  Ref: 9573190 J Bacteriol, 1998
- fecI encodes a sigma 70 type factor that is necessary for transcription of the ferric citrate transport genes fecABCDE.  **fecR**  encodes a cytoplasmic transmembrane protein that is required for the induction of fec transport gene transcription by ferric citrate binding to the  **FecA**  outer membrane receptor protein.  Ref: 9393439 Mol Gen Genet, 1997
- Three randomly generated inactive  **FecR**  mutants,  **FecR**  L254E,  **FecR**  L269G, and  **FecR**  F284L, were suppressed to different degrees by the mutants  **FecA**  G39R and  **FecR**  D43E.  Ref: 12813067 J Bacteriol, 2003
- The low resistance mediated by two  **FecR**  BlaM proteins in a  **fecA**  deletion mutant was increased 20 fold by transformation with a  **fecA**  encoding plasmid.  Ref: 9573190 J Bacteriol, 1998

**Non-interactive relationship** (e.g. studied together, co-existance, homology, etc.)

- An N terminally truncated, induction negative but transport active  **FecA**  protein did not bind to His 10  **FecR** .  Ref: 10633096 J Bacteriol, 2000
- The N terminus of  **FecA**  interacts in the periplasm with the C terminus of  **FecR** , through which the signal is transferred across the cytoplasmic membrane into the cytoplasm, where it increases the activity of the sigma factor Fecl, which then directs the RNA polymerase to the fec promoter upstream of  **fecA** .  Ref: 9044267 Mol Microbiol, 1997
- The degree of residual binding of  **FecR**  mutant proteins to  **FecA**  was correlated with the degree of transcription initiation in response to ferric citrate in the culture medium.  Ref: 12813067 J Bacteriol, 2003
- Constitutive expression caused by C terminally truncated  **FecR**  derivatives was not inhibited by the N terminal  **FecA**  fragment.  Ref: 9044267 Mol Microbiol, 1997
- One  **FecR**  mutant,  **FecR**  D138E, V197A, induced  **fecA**  promoter directed transcription constitutively in the absence of ferric citrate and bound more strongly than wild type  **FecR**  to  **FecA** .  Ref: 12813067 J Bacteriol, 2003
- All  **FecR**  BlaM proteins with at least 61  **FecR**  residues complemented a  **fecR**  mutant such that it could grow on ferric citrate as the sole iron source and induced  **fecA**  lacZ transcription independent of ferric citrate.  Ref: 9573190 J Bacteriol, 1998
- Bound ferric citrate does not have to be transported but initiates a signal that is transmitted by  **FecA**  across the outer membrane and by  **FecR**  across the cytoplasmic membrane into the cytoplasm, where the FecI extracytoplasmic function ECF sigma factor becomes active.  Ref: 11114913 J Bacteriol, 2001
- In this study, it was shown through the use of a bacterial two hybrid system that, in the periplasm, the C terminal  **FecR**  237 317 fragment interacts with the N terminal  **FecA**  1 79 fragment.  Ref: 12813067 J Bacteriol, 2003
- It is proposed that the information flux from the cell surface to the cytoplasm involves a series of conformational changes of the proteins  **FecA** ,  **FecR** , and FecI in that order.  Ref: 9148773 Arch Microbiol, 1997
- By analogy with the FecI  **FecR**   **FecA**  and PupI PupR PupB systems, our data suggest that bfrZ expression is inducible by binding of the cognate ligand to BfrZ and transduction of a signal through the envelope.  Ref: 11292812 J Bacteriol, 2001
- The in vivo assay involved the determination of the  **FecA** ,  **FecR** , and FecI interacting domains with the bacterial two hybrid Lex based system.  Ref: 10633096 J Bacteriol, 2000
- Three specific proteins are involved  **FecA**  in the outer membrane,  **FecR**  in the cytoplasmic membrane, and FecI in the cytoplasm.  Ref: 12732308 Curr Opin Microbiol, 2003
